# Prevalence of macular complications related to myopia – Results of a multicenter evaluation of myopic patients in eye clinics in France

**DOI:** 10.1111/aos.14246

**Published:** 2019-09-10

**Authors:** Nicolas Leveziel, Simon Marillet, Quentin Dufour, Olivier Lichtwitz, Yacine Bentaleb, François Pelen, Pierre Ingrand, Rupert Bourne

**Affiliations:** ^1^ University of Poitiers Poitiers France; ^2^ CHU Poitiers Poitiers France; ^3^ INSERM 1084 Poitiers France; ^4^ CIC 1402 Poitiers France; ^5^ Vision & Eye Research Unit Anglia Ruskin University Cambridge UK; ^6^ Public Health department University of Poitiers Poitiers France; ^7^ Point Vision Paris France

**Keywords:** atrophy, choroidal neovascularization, epidemiology, lacquer cracks, myopia, prevalence, retinoschisis, staphyloma

## Abstract

**Purpose:**

Uncorrected refractive errors are the first cause of vision impairment worldwide. High myopia is a frequent cause of sight‐threatening chorioretinal complications. The aim of this study was to evaluate the prevalence of macular complications, visual impairment and blindness in patients with myopia.

**Methods:**

A cross‐sectional multicenter study carried out in French eye clinics mainly dedicated to refractive errors. Myopia severity was defined as mild (−0.5 to −3 D), moderate (−3 to −6 D), high (−6 to −10 D) and very high (more than −10 D). Macular complications related to myopia included lacquer cracks, myopic choroidal neovascularization, chorioretinal atrophy and retinoschisis. The prevalences of macular complications, blindness and vision impairment were estimated with respect to degree of myopia and age. Eligibility criteria were myopia on the left eye of −0.5 D or more. Exclusion criteria included any missing data related to subjective refractive error, age, gender and any history of cataract or refractive surgery.

**Results:**

Data files from 198 641 myopic individuals with a mean age of 34 years (SD: 15 years) were analysed. The prevalence of mild, moderate, high and very high myopia was, respectively, 65.95%, 26.14%, 6.72% and 1.19%. The prevalence of macular complications in the high and very high myopia groups was 0.5% [0.39–0.64] and 4.27% [3.49–5.17]. The prevalence of blindness or vision impairment was observed in 10.10% [8.91–11.39%] of the very high myopic group. At 60 years old or over, the prevalences of blindness or vision impairment were, respectively, 9.75% [7.91–11.85%] and 25.71% [21.00–30.87%] in the high and very high myopia groups.

**Conclusions:**

This multicenter cross‐sectional study provides new insights in terms of prevalence of macular complications related to myopia. To our knowledge, this is one of the largest European studies focusing on individuals with myopia, particularly on the macular complications and the functional consequences in relation to myopia.

## Introduction

In 2000, according to definitions of myopia and high myopia by refractive error of −0.50 and −5 dioptres (D) or more, myopia and high myopia were estimated to affect 1.406 billion people (22.9%) and 163 million people (2.7%) in the world, respectively. In the same study, projections estimated that by 2050, the prevalence of myopia and high myopia will affect nearly 4.7 billion people and 1 billion people, respectively (Holden et al. 2016). The rise in prevalence has been documented extensively among young urban Asian populations, yet other populations are also concerned by the myopia epidemic. Indeed, between 1999 and 2004, the prevalence of myopia (defined by refractive error ≤−1D) in the United States among individuals aged 20 years or older rose from 20.5% to 36.2% (Vitale et al. 2008). From six studies providing refractive data on 29 281 individuals aged 40 years or older living in the US, Western Europe and Australia, the Eye Diseases Prevalence Research Group reported estimated prevalences of myopia (defined by refractive error ≤−1D) of 25.4%, 26.6% and 16.4%, respectively (Kempen et al. 2004).

In parallel, the prevalence of high myopia, usually defined as −5 or more or −6 dioptres or more of myopia, has increased, with prevalences approximating 20% in some young Asian populations (Lin et al. 2004; Koh et al. 2014).

Myopic maculopathy is a major cause of vision impairment worldwide. This spectrum of macular disorders usually associated with high and pathologic myopia frequently affects individuals at working age. In the Taizhou Eye Study conducted in China (2012–2013), myopic macular degeneration was the second cause of vision impairment in adults aged 45–59 years after cataract (Tang et al. 2015). In Japan, myopic maculopathy is also a major cause of blindness and vision impairment in persons aged over 40 years (Iwase et al. 2006). In the United States, Western Europe and Australia, macular degeneration, including age‐related macular degeneration (AMD) and myopic macular degeneration, is the major cause of blindness (Bourne et al. 2014).

Given a pandemic level of myopia, it is very likely that vision impairment related to myopic maculopathy will increase, with subsequent repercussions on socio‐economic cost, quality of life and working efficiency (Saw et al. 2005). Furthermore, the burden of myopic maculopathy in terms of disability‐adjusted life years (DALYs) is probably much higher than AMD, the first cause of vision impairment in developed countries, due to the age at occurrence of macular complications in high myopia. Indeed, lacquer cracks or myopic choroidal neovascularization frequently occur at around fifty years of age (Wolf et al. 2014; Ikuno et al. 2015), chorioretinal atrophy usually being the last stage of myopic maculopathy.

While many studies have focused on the prevalence of myopia itself and its consequence in terms of functional impact, the prevalence of macular complications of myopia and the functional consequences remain largely unknown.

In this context, we aimed to describe the prevalence of macular complications related to myopia and their visual impact in a large group of European individuals through a cross‐sectional study.

## Materials and Methods

### Study design

This cross‐sectional study was carried out in French eye clinics mainly dedicated to refractive errors.

### Data collection

Data collection included age, gender, subjective refractive error, visual acuity, any relevant medical history such as laser refractive surgery, cataract surgery and any macular complications related to myopia on both eyes. Best‐corrected visual acuity was assessed on a Monoyer chart and determined after objective auto‐refractometry followed by subjective refinement. All individuals underwent an ophthalmic examination, including non‐cycloplegic autorefraction on both eyes in adults (tono‐refractometer, Nidek^®^) and cycloplegic autorefraction with cyclopentolate for children, a slit‐lamp examination and a fundus examination.

The assessment of any macular complication related to myopia was made by an ophthalmologist, based on colour fundus images, auto‐fluorescence, SD‐OCT scans and fluorescein and indocyanin angiography if judged necessary by the physician himself.

Because grading of chorioretinal atrophy can be difficult in myopia, due to thinner choroid with maintained normal visual acuity, we only considered macular chorioretinal atrophy when the presence of patchy chorioretinal macular atrophy or macular atrophy was clearly mentioned in the data file.

### Refractive and examination data

Severity of myopia was classified as mild myopia (−0.5 to −2.75 D), moderate myopia (−3 to −5.75 D), high myopia (−6 to −9.75 D) and very high myopia (more than −10 D).

Macular complications related to myopia included lacquer cracks, past or active myopic choroidal neovascularization, chorioretinal atrophy and retinoschisis. Staphyloma was considered as an anatomical particularity of myopia rather than a complication. These different lesions were assessed by fundus examination, spectral domain optical coherence tomography (SD‐OCT) and fluorescein angiography if judged necessary by the ophthalmologist.

Exclusion criteria included any missing data related to subjective refractive error, age, gender, and any history of refractive or cataract surgery.

Vision impairment was defined using visual acuity (VA) for the better eye strictly less than 0.5–0.05 included. Blindness was defined as VA for the better eye strictly less than 0.05, according to the World Health Organization criteria.

Data collection was declared to the Commission nationale de l'informatique et des libertés (CNIL). The described research adhered to the tenets of the Declaration of Helsinki.

### Data annotation

The original data set contained the records of 602 103 myopic and non‐myopic patients.

In the initial database, information pertaining to complications and exclusion criteria was part of unstructured text fields. Patient annotation for a given complication was carried out through a three‐step text‐mining process:

- Extraction of relevant terms from all text fields
- Selection of records based on these terms (47 438 records)
- Manual review and annotation of the selected records

### Statistical analysis

A complication was assigned to a patient as opposed to an eye, that is regardless of whether the right or left eye was mentioned, it was considered present. Similarly, a patient was given visual acuity status (‘no vision impairment’, ‘vision impairment’ or ‘blindness’) based on the better eye. Refractive errors (REs), on the other hand, were assigned to an eye. Only left eyes were considered in the analysis for RE, making the common assumption that both eyes are highly correlated. Refractive errors were quantified by the sphere instead of the spherical equivalent.

The prevalence of vision impairment and blindness, and the prevalences of complications were computed with respect to myopia severity, age, and gender, and reported with exact Clopper–Pearson binomial 95% confidence intervals (CI).

Univariate and multivariate odds ratio (OR) for myopia severity were computed from logistic regression coefficients with corresponding Wald 95% CI.

The probability of macular complications as a function of the sphere was modelled using logistic regression, controlling for age and sex. Patients with sphere smaller than −17D were excluded because of low sample size. Model fit was checked using the Hosmer and Lemeshow test.

All analyses were performed with SAS/STAT software, version 9.4 of the SAS System for Windows. Copyright © 2016 by SAS Institute Inc.

## Results

### Demographic and refractive data

Data from 198 641 myopic patients (mean age 34 ± 15 years) were included in the analysis. The distribution of severity of myopia in this population was mild 65.95%, moderate 26.14%, high 6.72% and very high 1.19%. Demographic and refractive data are presented in Table 1.

**Table 1:** Age, refraction and functional data according to gender and myopia severity

	N (%)	Age Mean ± SD	Sphere Mean ± SD	Visual impairment % [95% CI]	Blindness % [95% CI]	Visual impairment or blindness % [95% CI]
Myopes	198 641 (100.00)	34 ± 15	−2.63 ± 2.21	0.54 [0.51–0.57]	0.53 [0.50–0.56]	1.07 [1.02–1.11]
Gender						
Females	110 777 (55.77)	34 ± 15	−2.73 ± 2.29	0.55 [0.50–0.59]	0.52 [0.48–0.56]	1.06 [1.00–1.12]
Males	87 864 (44.23)	35 ± 15	−2.50 ± 2.10	0.53 [0.48–0.58]	0.54 [0.49–0.59]	1.07 [1.00–1.14]
Myopia class						
Mild myopia	131 001 (65.95)	34 ± 15	−1.41 ± 0.70	0.37 [0.33–0.40]	0.53 [0.49–0.57]	0.89 [0.84–0.95]
Moderate myopia	51 920 (26.14)	35 ± 14	−4.05 ± 0.82	0.45 [0.39–0.51]	0.49 [0.43–0.56]	0.94 [0.86–1.03]
High myopia	13 355 (6.72)	36 ± 14	−7.20 ± 1.03	1.21 [1.03–1.41]	0.44 [0.34–0.57]	1.65 [1.44–1.88]
Very high myopia	2365 (1.19)	41 ± 15	−13.00 ± 3.31	8.44 [7.34–9.63]	1.66 [1.18–2.26]	10.10 [8.91–11.39]

Myopia severity in the left eye was defined as mild (−0.5 to −3 D), moderate (−3 to −6 D), high (−6 to −10 D) and very high (more than −10 D). Vision impairment and blindness were defined as best‐corrected visual acuity (VA) for the better eye strictly less than 0.5–0.05 included and strictly less than 0.05, respectively.

### Macular complications

In terms of macular complications, the prevalence of lacquer cracks (LC) and myopic choroidal neovascularization (mCNV) was, respectively, 0.07% [0.03–0.13%] and 0.07% [0.03–0.13%] in the high myopia group, whereas these frequencies were 0.51% [0.26–0.88%] and 0.42% [0.20–0.78%]) in the very high myopia group. The prevalence of chorioretinal atrophy (CRA) was 0.39% [0.29–0.51%] and 3.42% [2.73–4.24] in the high and very high myopia groups, respectively. Similarly, the prevalence of retinoschisis was 0.03% [0.01–0.08%] and 0.30% [0.12–0.61%]. These results are detailed in Table 2.

**Table 2:** Prevalence of staphyloma and macular complications, and odds ratios, with respect to myopia severity

		Mild myopia (N = 131 001)		Moderate myopia (N = 51 920)		High myopia (N = 13 355)		Very high myopia (N = 2365)	
Staphyloma	N, % [95% CI]	136	0.10 [0.09–0.12]	260	0.50 [0.44–0.57]	269	2.01 [1.78–2.27]	181	7.65 [6.61–8.80]
	OR [95% CI]	Reference		4.94 [4.02–6.09]		19.42 [15.78–23.90]		63.95 [50.85–80.43]	
Macular complications	N, % [95% CI]	56	0.04 [0.03–0.06]	72	0.14 [0.11–0.17]	67	0.50 [0.39–0.64]	101	4.27 [3.49–5.17]
	OR [95% CI]	Reference		3.43 [2.42–4.87]		11.72 [8.20–16.75]		74.31 [53.20–103.79]	
Chorioretinal atrophy	N, % [95% CI]	44	0.03 [0.02–0.05]	57	0.11 [0.08–0.14]	52	0.39 [0.29–0.51]	81	3.42 [2.73–4.24]
	OR [95% CI]	Reference		3.48 [2.35–5.17]		11.66 [7.78–17.46]		74.08 [50.94–107.75]	
Lacquer cracks	N, % [95% CI]	3	0.00 [0.00–0.01]	4	0.01 [0.00–0.02]	9	0.07 [0.03–0.13]	12	0.51 [0.26–0.88]
	OR [95% CI]	Reference			3.52 [0.79–15.76]	29.09 [7.86–107.64]		158 [44.39–565.51]	
Myopic choroidal neovascularization	N, % [95% CI]	5	0.00 [0.00–0.01]	6	0.01 [0.00–0.03]	9	0.07 [0.03–0.13]	10	0.42 [0.20–0.78]
	OR [95% CI]	Reference		3.14 [0.96–10.32]		17.00 [5.68–50.90]		70.60 [23.98–207.85]	
Retinoschisis	N, % [95% CI]	7	0.01 [0.00–0.01]	9	0.02 [0.01–0.03]	4	0.03 [0.01–0.08]	7	0.30 [0.12–0.61]
	OR [95% CI]	Reference		3.29 [1.22–8.84]		5.37 [1.57–18.35]		40.56 [14.07–116.90]	

Odds ratios (adjusted for age and gender) of moderate, high and very high myopia for the occurrence of complications were computed with logistic regression and use mild myopia as reference. Myopia severity in the left eye was defined as mild (−0.5 to −3 D), moderate (−3 to −6 D), high (−6 to −10 D) and very high (more than −10 D).

**Figure 1:**
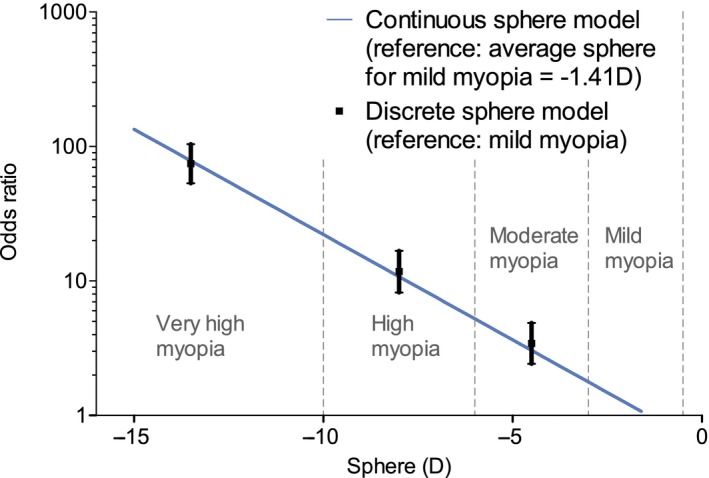
Logistic model of the risk to present macular complications with respect to myopia degree. Macular complications include lacquer cracks, myopic choroidal neovascularization, chorioretinal atrophy and retinoschisis. Myopia severity in the left eye was defined as mild (−0.5 to −3 D), moderate (−3 to −6 D), high (−6 to −10 D) and very high (more than −10 D).

A significant increase in the prevalence of macular complications in relation to the severity of myopia was observed. After correcting on age and gender, the probability of any macular complication depends exponentially on the sphere, with odds ratio for a unit dioptre decrease equal to 1.43 [1.40–1.47]. In other words, a decrease by one dioptre results in a risk multiplied by 1.43, age and sex remaining equal (Fig. 1).

Taking mild myopia as the reference group, univariate ORs for lacquer cracks were 29.45 [7.97–108.78] and 223.69 [62.80–789.65] in the high myopia and very high myopia groups. Similarly, the ORs for myopic choroidal neovascularization were 17.67 [5.92–52.73] and 111.25 [38.00–325.73], respectively. Considering chorioretinal atrophy, risk was associated with an odds ratio of 11.63 [7.78–17.39]) and 105.55 [72.95–152.72] in the high myopia and very high myopia groups, respectively.

**Figure 2:**
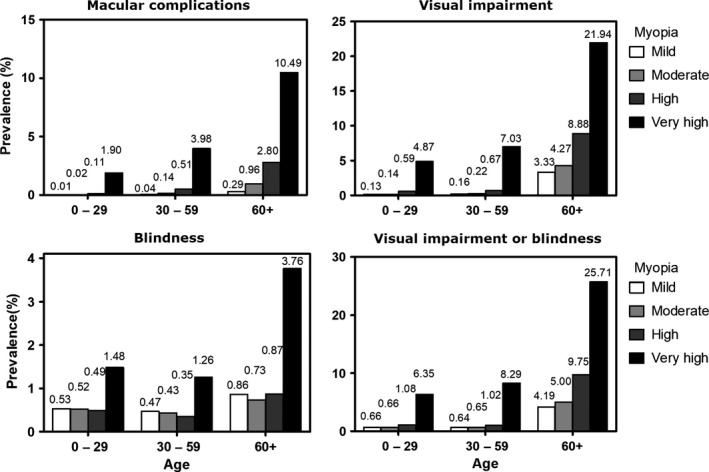
Prevalences of macular complications, vision impairment and blindness depending on age and myopia severity. Macular complications include lacquer cracks, myopic choroidal neovascularization, chorioretinal atrophy and retinoschisis. Myopia severity in the left eye was defined as mild (−0.5 to −3 D), moderate (−3 to −6 D), high (−6 to −10 D) and very high (more than −10 D). Vision impairment and blindness were defined as best‐corrected visual acuity (VA) for the better eye strictly less than 0.5–0.05 included and strictly less than 0.05, respectively.

After adjustment for age and gender, multivariate logistic regression analysis showed that risk of lacquer cracks was associated with ORs of 29.09 [7.86–107.64] and 158.45 [44.39–565.51] in the high myopia and very high myopia groups, respectively. Similarly, the ORs for myopic choroidal neovascularization were 17.00 [5.68–50.87] and 70.60 [23.98–208.85]. Considering chorioretinal atrophy, risk was associated with an odds ratio of 11.66 [7.78–17.46] and 74.08 [50.94–107.75] in the high myopia and very high myopia groups. These results are detailed in Table 2, while the prevalence of macular complications depending on age and myopia severity is presented in Figure 2.

### Functional impact

Vision impairment and blindness were observed in 0.54% [0.51–0.57%] and 0.53% [0.50–0.56%], respectively, of the myopic population. In the high myopia group, frequencies of vision impairment and blindness were 1.21% [1.03–1.41%] and 0.44% [0.34–0.57%], respectively, whereas these frequencies were 8.44% [7.34–9.63%], and 1.66% [1.18–2.26%]) in the very high myopia group.

Subgroup analyses of vision impairment and blindness were also performed by defining three subgroups categorized by the 0–29 years, 30–59 years and 60 years old or more groups. In the 30–59 years age group, the prevalences of blindness or vision impairment were 1.02% [0.80–1.29%] and 8.29% [6.87–9.89%], respectively, in the high and very high myopia groups. In the 60 years old or more age group, the prevalences of blindness or vision impairment were 9.75% [7.91–11.85%] and 25.71% [21.00–30.87%], respectively. These results are detailed in Fig. 2.

## Discussion

The main objective of the present study was to investigate the macular complications of myopia and associated vision impairment in a large group of individuals. In this group of individuals with a mean age of 34 years, vision impairment or blindness was observed in 1.65% [1.44–1.88] and 10.10% [8.91–11.39] of high and very high myopic patients (Table 1). These results, for a rather young group of European patients, obviously raised the question of visual impact of myopia in older populations and in populations with a higher incidence of high myopia. This concern is confirmed by the fact that of all the persons with high myopia and very high myopia reported in this study, 9.75% [7.91–11.85%] and 25.71% [21.00–30.87%], respectively, had developed vision impairment or blindness by the age of 60.

### Literature data

These results are corroborated by Tideman et al. In a cross‐sectional study based on 15 693 Europeans drawn from population‐based data in the Rotterdam Study I to III, the Erasmus Rucphen Family Study and from case–control data in the Myopia Study, all of them from the Netherlands, the cumulative risk of vision impairment (best‐corrected VA in the right eye strictly less than 0.3–0.05 included) by age 75 years was 20.0% for −6 to greater than −10 D, 19.9% for −10 to greater than −15 D, and 80.3% for −15 D or less (Tideman et al. 2016).

In a Scandinavian study including 10 135 participants randomly selected by the Copenhagen central population registry from a prospective cardiovascular population‐based study, myopia‐related retinal disorders accounted for 7% of the causes of vision impairment (best‐corrected VA in the better eye worse than 0.5 and better than 0.01) and for 14% of causes of blindness (best‐corrected VA in the better eye equal to 0.01 or worse) in the total study population. However, between 20 and 64 years of age, myopia‐related retinal disorders accounted for 26% of the causes of vision impairment and for 14% of the causes of blindness (Buch et al. 2004).

Previously, the Rotterdam study showed that myopic degeneration was a major cause of vision impairment in subjects younger than 75 years (Klaver et al. 1998).

In a Chinese population‐based cohort study including 10 234 participants aged 45 years or more, the first cause of total bilateral and monocular vision impairment among adults 45–59 years of age was myopic macular degeneration in 59.6% and 27.2%, respectively (Tang et al. 2015). In other studies on Chinese populations, the Beijing Eye Study and the Shihpai Taiwan Eye Study, myopic macular degeneration accounted for 32.7% of low vision and 12.5% of vision impairment, respectively (Hsu et al. 2004; Xu et al. 2006).

Even if clearly demonstrated, the precise relationship between degree of myopia and risk of developing macular complications remains to be clarified. There may be a relationship with increased risk of complications for each unit increase in spherical equivalent in dioptres and/or unit increase in axial length in millimetres and a threshold beyond which the risks of complications increase exponentially after a certain level of refractive error.^19^ In the current study, due to large sample size, we observed that the risk of macular complication increased exponentially with myopia degree.

### Myopia and vision impairment

Overall vision impairment due to myopia obviously combines both refractive error itself and disabilities due to ocular complications which mainly include cataract, glaucoma, macular complications and retinal detachment. It is likely that the burden of uncorrected refractive error itself represents the main part of DALYs related to myopia in countries with a low socio‐economic level, whereas complications of myopia are likely to represent the main part of DALYs in developed countries where access to optical corrections by lenses, glasses or by refractive surgery is more widely available. Interestingly, in a systematic review based on surveillance of the prevalence and causes of vision impairment in high‐income countries and Central/Eastern Europe, uncorrected refractive error was the leading cause of moderate and severe vision impairment defined by visual acuity in the better eye of worse than 6/18 to 3/60 inclusive, contributing to almost half of the vision impairment burden (Bourne et al. 2018).

### Limitations

We acknowledge some limitations of this study. Although the sample size was large, caution should be taken in interpretation of the frequencies reported in this study, because this was a clinic‐based sample mainly serving people with refractive errors, and therefore is not necessarily representative of what would be found in the general population. Differences between clinic‐based and population‐based findings may be less notable when considering those aged 50 years or more in our sample as a higher proportion of the population in this age is likely to seek optometric assessment for reasons of presbyopia.

Patients with a history of cataract or refractive surgery were excluded because their refractive status prior to surgery was unknown. Since 1934 patients (0.97%) were in this situation, these exclusions may have slightly lowered the overall frequency of vision impairment.

Furthermore, while the cross‐sectional design of this study provided much information on the visual function and macular complications at a given time, the temporal sequence of the macular complications cannot be determined by this approach. In the same way, myopic eyes with vision impairment are frequently prone to have several macular complications, so that it is not possible to be absolutely confident of the principal contributing factor to vision impairment. For this reason, we did not attempt to correlate any particular macular complications to visual function.

The international photographic classification and grading system of myopic maculopathy according to the META‐analysis for Pathologic Myopia study group defined five categories of myopic maculopathy, including no myopic retinal change (category 0), tessellated fundus (category 1), diffuse chorioretinal atrophy (category 2), patchy atrophy (category 3) and macular atrophy (category 4) with additional features defined as ‘plus’ lesions (lacquer cracks, myopic choroidal neovascularization and Fuchs spot) (Ohno‐Matsui et al. 2015). The current study only focused on the last two categories of chorioretinal atrophy and did not consider tessellated fundus or diffuse atrophy because these two forms of atrophy, according to the study design, were considered as less clinically relevant under the scope of vision impairment and blindness than the other two, more advanced forms of atrophy.

The choice of a classification of myopia according to the degree of refractive error can also be challenged, because it has been clearly demonstrated that no safe threshold for myopic refractive errors exists. Indeed, macular complications of myopia are frequently observed even below −6 D in absolute value (Flitcroft 2012). We chose to follow a convention of categorizing myopes by refractive error magnitude in common with many other studies. It may also be objected that pathologic macular changes are better correlate with axial length than with refractive errors. However, the current data reflect the daily ophthalmological practice of French ophthalmologists and axial length measurements are not currently recommended for usual follow‐up of myopic patients. Furthermore, it has been shown that although slightly lower cumulative risks of visual impairment in myopia are observed when using refractive data, the trends are similar when using axial length (Tideman et al. 2016).

### Myopia and strategies to deal with it

Myopia has been defined as one of the five immediate priorities for the ‘Vision 2020’ initiative by the World Health Organization because it is a major cause of vision impairment in populations throughout the world (Pararajasegaram 1999). In this context, two complementary approaches are needed. It is of crucial importance to investigate more precisely the impact of all potential complications of myopia on visual acuity on targeted populations. The present study focused on the macular complications of myopia and not on other ocular complications related to high myopia, such as cataract, optic neuropathy and retinal detachment, which are frequently associated with myopic maculopathy. Analyses of the data specifically related to retinal detachment and glaucoma are ongoing.

Secondly, given the global increase of myopia and high myopia, there is a need to develop nationwide preventive strategies. Some Asian countries confronted with the highest myopia incidence are developing such strategies, based on environmental, pharmacologic and optical approaches targeting the two pathways for myopia control: slowing the onset of myopia and reducing or preventing progression. For example, it has been demonstrated that longer outdoor time can be of benefit on myopia onset but not on myopia progression (Jones‐Jordan et al. 2012; French et al. 2013).

Comparison of studies aiming to evaluate the impact of near‐work activities on myopia is a challenge since near‐work activities (such as studying, reading, computer use or watching TV) are diversely defined and because studies have reported highly different outcomes regarding progression of myopia. While some studies conclude that near‐work activities are a risk factor, others do not (Wu et al. 2010, 2015; Lee et al. 2015).

In a systematic review and meta‐analysis, association between near‐work activities and myopia indicated a 2% increase in the odds of myopia occurrence per additional dioptre‐hour of time spent on near work per week (Huang et al. 2015).

Children treated by atropine 0.01% have lower myopia progression with minimal myopic rebound after atropine is stopped and present negligible effects on accommodation, pupil size and near visual acuity (Chia et al. 2012, 2014, 2016). Its mechanism of action remains to be specified.

Orthokeratology uses reverse geometry lenses worn overnight to temporarily flatten the corneal centre and to provide a peripheral myopic defocus. A meta‐analysis including 7 studies showed that this approach results in 45% reduction of myopia progression at 2 years (Sun et al. 2015). However, its indications must be discussed for each case according to the degree of myopia (mild or moderate), risk of discomfort and microbial keratitis.

Other therapeutic strategies to slow down myopia progression have been evaluated: undercorrection of myopia is not effective and may potentially be harmful (Chung et al. 2002), and rigid gas oxygen‐permeable lenses do not slow myopia progression (Katz et al. 2003).While many approaches aimed at decreasing myopia progression presently exist, there remains a need to define precisely preventive personalized protocols for each myopic patient, depending mainly on degree of myopia, age and socio‐economic integration.

In summary, this is to our knowledge the widest survey of prevalence of myopia in Europe aiming to investigate macular complications and their visual impact. We hope that the sight‐threatening impact of myopia will lead to future therapeutic personalized protocols stratified by age group and degree of myopia.
